# Stiffness-tunable velvet worm–inspired soft adhesive robot

**DOI:** 10.1126/sciadv.adp8260

**Published:** 2024-11-20

**Authors:** Hyeongho Min, Daebeom Bae, Siyeon Jang, Sangmin Lee, Myungjin Park, Cem Balda Dayan, Jiwoong Choi, Keungyonh Bak, Yoosoo Yang, Sungwoo Chun, Metin Sitti

**Affiliations:** ^1^ Physical Intelligence Department, Max Planck Institute for Intelligent Systems, 70569 Stuttgart, Germany.; ^2^ Department of Electronics and Information Engineering, Korea University, 30019 Sejong, Republic of Korea.; ^3^ Institute for Biomedical Engineering, ETH Zurich, 8092 Zurich, Switzerland.; ^4^ Department of Electronics and Computer Engineering, Hanyang University, 04763 Seoul, Republic of Korea.; ^5^ School of Integrated Technology, Gwangju Institute of Science and Technology, Gwangju, Republic of Korea.; ^6^ Medicinal Materials Research Center, Biomedical Research Division, Korea Institute of Science and Technology, 02792 Seoul, Republic of Korea.; ^7^ School of Medicine and College of Engineering, Koç University, 34450 Istanbul, Turkey.

## Abstract

Considering the characteristics and operating environment of remotely controlled miniature soft robots, achieving delicate adhesion control over various target surfaces is a substantial challenge. In particular, the ability to delicately grasp wrinkled and soft biological and nonbiological surfaces with low preload without causing damage is essential. The proposed adhesive robotic system, inspired by the secretions from a velvet worm, uses a structured magnetorheological material that exhibits precise adhesion control with stability and repeatability by the rapid stiffness change controlled by an external magnetic field. The proposed adhesion protocol involves controlling soft-state adhesion, maintaining a large contact area, and enhancing the elastic modulus, and the mechanical structure enhances the effectiveness of this protocol. Demonstrations of the remote adhesive robot include stable transportation in soft and wet organs, unscrewing a nut from a bolt, and supporting mouse tumor removal surgery. These results indicate the potential applicability of the soft adhesive robot in biomedical engineering, especially for targeting small-scale biological tissues and organisms.

## Teaser

A stiffness-tunable adhesive robot attaches to soft objects using structured magnetorheological materials.

## INTRODUCTION

Recent research on miniature mobile robots with remote control and advanced mobility is promising for minimally invasive medical operations, including disease diagnostics, treatment, triggered and local drug delivery, and surgery (*1*). Among these advances, the often-underestimated adhesive control of miniature robots is one of the most crucial aspects of their functionality, serving as the cornerstone for practical tasks in diverse environments, such as inside the human body on rough, textured, and soft tissues. In addition, on a small scale, the issues associated with adhesion, friction, and surface energy grow exponentially, making it a continuous challenge to manipulate objects or for robots to stably fix and move within the human body. Continuous research is crucial for developing a reversible, simple, and reliable adhesive control mechanism for practical applications of miniature robots in challenging biological environments.

Artificial systems for adhesion control have traditionally been inspired by the effective adhesive strategies of natural organisms specialized in survival strategies, such as mussel proteins (*2*), gecko (*3*–*9*), frog (*10*), beetle foot hairs (*11*), and octopus suckers (*12*). In particular, for switchable adhesives, there has been a preference for studies that control nonchemical adhesion parameters, which are easier to handle, faster, and harmless, rather than chemical adhesion, which involves high energy consumption for detachment. Specifically, using reversibly controllable energy sources, such as light (*13*, *14*), heat (*15*), magnetic field (*16*–*20*), and pressure (*21*, *22*), based on theoretical analyses of mechano-dynamics, attempts have been predominant to control effective adhesion through changes in contact area (*23*–*25*), interfacial transitions (*26*), energy distribution (*27*, *28*), pneumatic alterations (*29*), modulus reinforcement (*30*), and electrostatics (*31*), as well as through the design of three-dimensional (3D) micro/nanostructures (*18*, *20*). Elastic modulus is considered the most important factor in microscale adhesion systems because it affects conformal surface contact and mechanical strength, which are essential for adhesion. Considering the operational environment for robotics, such as damage to biological systems and varying surface roughness and shapes, an approach based on the elastic modulus is appropriate, but the adhesive mechanism must be designed to take into account the efficiency of external energy transmission, as well as speed and reversibility for the robot’s adhesion control.

**Fig. 1.**
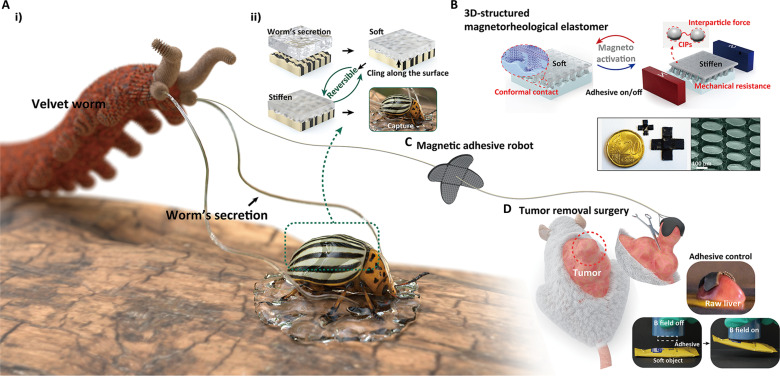
Bioinspired adhesion-controlled soft millirobot with its adhesion mechanism and applications. (**A**) (i) Schematic diagram of a velvet worm that captures the prey by secretions with modulated modulus. (ii) Capturing mechanism of the secretion: sticking to the surface of the prey in its soft state, and then hardening for immobilizing the prey. (**B**) Schematic and scanning electron microscopy (SEM) images of the 3D structured magnetorheological elastomer (MRE) that modulates elastic modulus by the external magnetic field for adhesion control. CIPs, carbonyl iron particles. (**C**) Schematic and optical images of the designed magnetically responsive adhesive robot with an MRE adhesive. (**D**) Mouse tumor removal surgery in vivo with the assistance of a remote adhesive robot with the optical images of gripping soft and easily deformable objects by MRE adhesive and robot.

Nature has found an elegant solution for switchable adhesive mechanisms to solve this issue. Animals, such as centipedes (*32*), frogs (*33*), and snails (*34*), control the elastic modulus of their saliva through methods, such as solvent evaporation, ion concentration regulation, and the application of shear stress, enabling them to hunt prey or move. On the basis of this inspiration, studies on switchable adhesion using shape memory polymers (SMPs), which change modulus due to temperature, have shown great potential (*30*, *35*). However, SMPs relying on heat as an energy source face issues, such as time-dependent behavior, thermal stability, manufacturing complexity, difficulties with structuring, and thermal transfer effect, which hinder their effectiveness for remote robotic systems. The velvet worm, belonging to the Peripatidae family, uses a slightly different mechanism, using a strategy of shooting and solidifying a soft secretion for hunting (Fig. 1A). This secretion uses a unique mechanism, where nanoglobules dispersed within the secretion self-align into nano- and microfibrils under mechanical stress, resulting in an enhancement of the elastic modulus (*36*, *37*). We can find a close connection in magnetorheological (MR) materials, which adjust their rheological properties through the alignments and attraction forces between magnetic particles enhanced by an external magnetic field (*38*). This magnetic field–driven material can induce rapid rheological changes, offering powerful advantages such as fine control, remote operation, exceptional stability, and quick response. Several studies on grippers using the dispersion of MR materials in fluids to trigger viscosity transformations (*24*, *39*–*41*) and hybrid materials mixed with elastomers using pneumatics (*42*) have been reported. Despite their impressive performance, this system faces limitations in miniature robots or industrial applications owing to their fluidic properties, such as fluidic-rheological behavior, fluid evaporation, and nonplanar structuring. Moreover, a theoretical modeling of their adhesive mechanism is unavailable.

This paper presents a remotely controllable magnetic adhesive robot inspired by the velvet worm, fused with a mechanical structure made of MR elastomers (MREs). MREs are composed of 3D networks of magnetic micro/nanoparticles and an elastomeric matrix, where the mobility of the polymer chains is restricted by the increasing magnetic forces among the particles under a strong magnetic field, enhancing the stiffness of the elastomer, as shown in Fig. 1B. Such materials can be used to grip objects with varying surface roughness and stiffness across multiple length scales and easily switch between on/off states through remote magnetic field–based triggering. Furthermore, a material modeling and design strategy is proposed to optimize the control of adhesive forces with elastic modulus and surface energy conversion due to the magnetic field. External magnetic fields are used to control the stiffness state [elastic modulus: 0.477 MPa (at 0 T) and 2.03 MPa (at 0.36 T)], achieving stable grasping (~2.93 N/cm^2^) on porcine tissue replicas. MRE-based mushroom-shaped 3D structures expand the range of possible gripped objects and effectively reduce the required preload (~0.5 N/cm^2^), solving deformation or damage issues for fragile objects and the limitations of the low forces of miniature robots (Fig. 1C). Last, practical gripping applications are demonstrated, such as precise and stable grasping and transportation of various fragile objects, including soft tofu, wet salmon roe, and slippery wet organs (Fig. 1D), and assisting in the removal surgery of mouse tumors (Fig. 1E) using the proposed miniature adhesive robot.

## RESULTS

### Optimization of the MRE for adhesion control

**Fig. 2.**
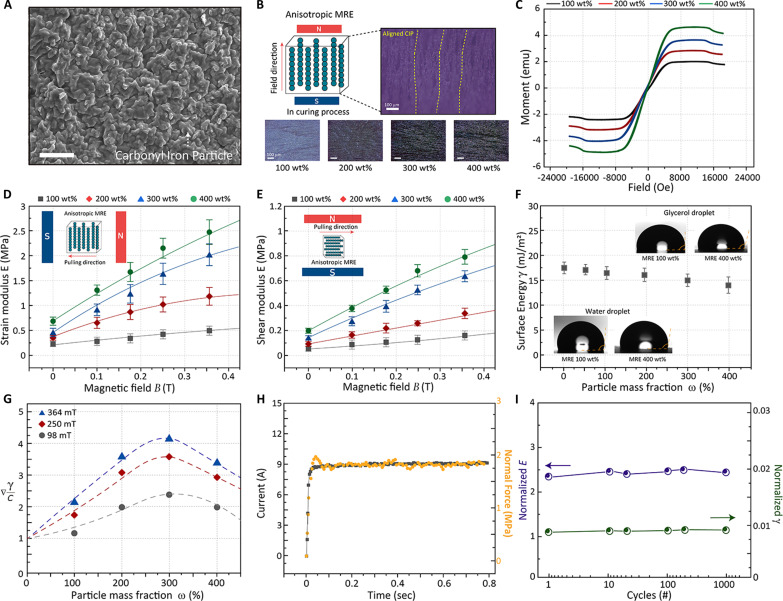
Characterization of the optimized MRE sheet for adhesive control. (**A**) SEM images of MRE composite. Scale bar, 25 μm. (**B**) Schematic and optical images of the MRE sheet surface with aligned CIP fillers in different mass ratio conditions. (**C**) Magnetic hysteresis loop of the MRE sheet due to different ratios of the CIP fillers. (**D** and **E**) Analysis of mechanical properties (tensile modulus and shear modulus) of MRE according to the filler mass ratio under a magnetic field. (**F**) Analysis of the MRE surface energy change according to the composition ratio with water and glycerol contact angle. (**G**) Optimizing the particle mass fraction of MRE, a characteristic quantity that determines the maximum change in adhesive forces under the applied magnetic field. (**H**) Response time between the current control for applying magnetic field and modulus change of MRE. (**I**) Durability assessment of MRE’s mechanical properties through repeated testing (1000 times).

The magnetically triggered MRE stands out as an adhesive material for the gripper because of its exceptional magnetic responsiveness. This section describes the material design strategy for MRE, which is tailored to have suitable magnetic responsiveness, surface energy, time-dependent behavior, and repeatability as an adhesive material for an adhesive robot. Soft magnetic materials as fillers with high magnetic permeability are traditionally used to improve the magneto-responsiveness of MRE. On the other hand, when using highly elastic polymers, such as polydimethylsiloxane (PDMS) or Dragon Skin, as the matrix, the initial high modulus notably reduces the magnetic responsiveness due to the low influence of the magnetic microparticles owing to the initial elasticity (fig. S1). Therefore, the MRE composite was constructed using soft magnetic particles, carbonyl iron particles (CIPs; diameter: ~5 μm) and Ecoflex-10, a highly soft elastomer. In addition, the problem of CIP agglomeration was addressed using a simple composite mixing process, ensuring proper dispersion and minimal porosity, as confirmed by scanning electron microscopy (SEM) (Fig. 2A). The issue of CIPs settling within the matrix was mitigated by minimizing it through additional thermal curing, resulting in rapid polymer curing (fig. S2). MRE also exhibits anisotropic properties in which the MR performance varies according to the internal CIP alignment (*43*). A magnetic flux triggered by an external magnetic field determines the alignment direction of the particles within the matrix before the matrix cures (fig. S3). Consequently, a uniaxial alignment of CIPs during matrix curing enhances the anisotropic magnetic response of MRE (Fig. 2B, experimental details in note S2, and anisotropic property in fig. S4).

The filler loading concentration was designed to optimize the adhesive control performance based on the internal structural formation of fillers in this composite. In particular, this paper emphasizes the specific factors of elastic modulus and surface energy changes, which are critical elements in adhesion. Figure 2C shows that the magnetic moment change of MRE becomes more substantial as the CIP ratio increases. Correspondingly, higher filler composition ratios lead to an increased change in tensile modulus (also, shear modulus that affects shear adhesion) (*44*, *45*) with notable magnetic field changes (100 wt %: 0 T–0.22 MPa, 364 mT–0.52 MPa; 200 wt %: 0 T–0.278 MPa, 364 mT–1.06 MPa; 300 wt %: 0 T–0.477 MPa, 364 mT–2.03 MPa; 400 wt %: 0 T–0.746 MPa, 364 mT–2.46 MPa), as shown in Fig. 2 (D and E). On the other hand, an increase in fillers increases the initial modulus, which leads to a decrease in the change rate of the material modulus (*∆E*/*E*_0_) [(*∆E*/*E*_0_: 4.33 at 200 wt %, 4.2 at 300 wt %, and 3.28 at 400 wt %) between 0 and 0.364 T] (*46*). Furthermore, an investigation of changes in surface energy, one of the important adhesion parameters, is needed (Fig. 2F and fig. S5). The surface energy can be determined using glycerol and the contact angle of a water droplet and corresponding theoretical calculations based on the Owen-Wendt equation (details in note S1) (*47*). The surface energy decreases as the filler ratio increases, but the rate of change according to the magnetic field slightly increases (fig. S6). These two adhesion parameters, surface energy (γ) and elastic modulus $\left(\frac{1}{C}\right)$, can be interpreted as standardized adhesive control strength by the quantity $\frac{\gamma }{C}$. Assuming that both *C* (adhesive compliance, $\frac{1}{E}$) and γ depend on the particle mass fraction, Eq. 1 implies the possibility of an optimal point where the value of normalized adhesive force (*F_n_*) conversion is maximized as (*48*, *49*)

$$F_{n}∽\sqrt{\frac{\gamma }{C}}$$ (1)

According to the experimental results, the critical mass fraction showing the highest adhesive change of the material is measured as the ratio (300 wt %) (Fig. 2G). The time required for the modulus shift in MRE (<60 ms) was examined to validate its suitability as a rapid and precise gripper material, as shown in Fig. 2H. The tuned elastic modulus time was compared according to the current control time because the ultimate magnetic control method of a robot would be by electromagnets or a Helmholtz coil. Moreover, the durability of a gripping material was verified by examining the mechanical conversion rates and changes in surface energy under repetitive magnetic field conditions (1000 times) (Fig. 2I).

### Adhesive analysis of 3D structured MRE adhesive

**Fig. 3.**
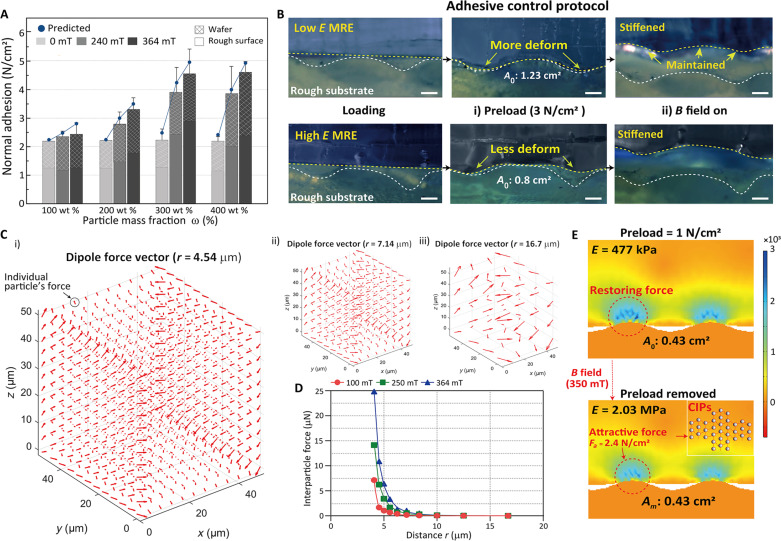
Adhesive control protocol of MRE and the analysis. (**A**) Adhesive measurement of the flat MRE adhesive (according to the composition ratio and substrate roughness). (**B**) Optical images showing adhesive control protocol with differences of contact area under preload with the enhanced stiffness by the magnetic field according to the composition ratio. Scale bars, 100 μm. (**C**) Finite element analysis (FEM) analysis of vector values of interparticle forces according to the particles’ distance, which depends on the composition of MRE [(i) 400 wt % MRE, (ii) 100 wt % MRE, and (iii) 25 wt % MRE]. (**D**) Calculated magnitude of interparticle force due to the distance of particles by different composition ratios and magnetic fields. (**E**) FEM analysis of the adhesive force with an understanding of the related elements (restoring force, attractive force, and contact area).

This section reports a detailed analysis of the adhesion control on objects with varying surface roughness and stiffness using the 3D structured MRE and various composition ratios, including theoretical modeling. Figure 3A (shear adhesion in fig. S7) shows the adhesive forces of MRE with various composition ratios caused by a change in magnetic field and substrates. Adhesive control protocol using MRE proceeds (MR adhesive process) as follows. (i) In the absence of an external magnetic field, the MRE adhesive, in its pliable state, adheres to the target with a sufficient preload, ensuring a broad contact area to the object (*A*_0_). (ii) As the external magnetic field increases in this state, interparticle forces restrict the restoration force of the polymer, maintaining a wide *A*_0_, and the *E* of MRE increases, resulting in higher adhesive strength. For a wafer, as the contact area before and after preload remains relatively constant, the improvement in adhesive force with increasing external magnetic field is influenced only by the increase in modulus. Although the increased adhesion becomes noticeable with an increase in filler concentration, the tendency is saturated because of the decrease in surface energy. On rough surfaces, however, during the preload process for adhesive, the deformation of MRE occurs along the wrinkles, which alters the polymer restoration force and the initial contact area. In this process, the increased attractive force of CIPs, driven by the magnetic field, restricts the polymer restoration force, preventing changes in the contact area with the adhesive force by the elevated elastic modulus. Unlike the case of a wafer substrate, in this scenario, 300 wt % MRE exhibited the highest adhesion and adhesion change rate, showing distinct differences in the initial modulus-dependent contact area, along with a clear limitation of polymer restoration (see Fig. 3B with MRE adhesive control protocol). Unlike previous studies in MR, our research using MRE is advantageous in terms of theoretical approach and validation. Finite element analysis (FEM)–based numerical simulations and equations were conducted for comparing the restoring force of elastomer (*F_r_*) and interparticle attractive force (*F_a_*) to gain a comprehensive understanding of the MRE adhesive control process, also including the adhesive elements as contact area (*A*), elastic modulus (*E*) (fig. S8), and adhesive force (*F*_*N*, *M*_). Figure 3 (C and D) shows the dipole force vector and its magnitude of individual particles’ attractive force in MRE under different magnetic fields and the distance of particles. In addition, the restoring force is investigated under the preload on MRE, as shown in Fig. 3E. These results highlight the essential need for superior conformal contact in high-elasticity adhesive structures to separate nonflat surfaces for greater adhesive strength, confirming the superior contribution of the controlled modulus to the mechanical detach with the theoretical equation in Discussion.

**Fig. 4.**
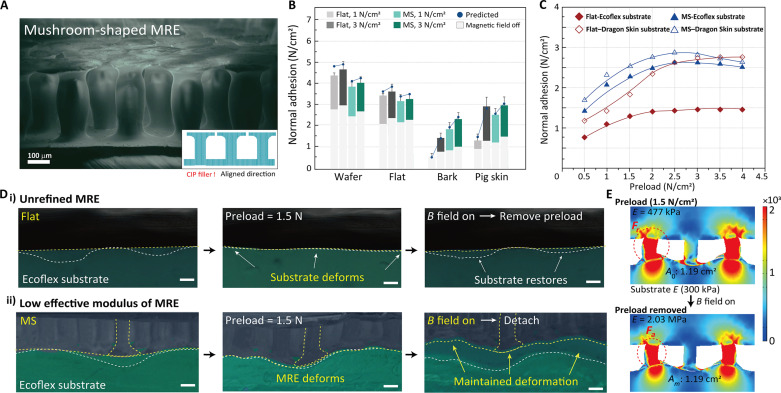
Adhesive control of the 3D structured MRE on soft material. (**A**) SEM image and schematic image of the 3D structured MRE with aligned CIP fillers. (**B**) Measurement of the adhesive force of flat and the 3D structured MRE adhesive according to the change in substrate roughness (Dragon Skin) and preload with magnetic field on/off. (**C**) Measurement of the adhesion force of the MRE adhesive according to the structure, preload force, and stiffness of the subject on a pig skin replica. (**D**) Sequential images of the MR adhesive process according to the structure. (i) Deformation of the substrate under the adhesive control protocol by flat MRE. (ii) Deformation of a 3D structured MRE with low effective modulus under adhesive control protocol. Scale bars, 100 μm. (**E**) FEM analysis of restoring force and the contact area of the 3D structured MRE adhesive on a softer surface.

Along with this MR effect, the 3D structure of the adhesive increases the applicability of the gripper to the soft and wrinkled surface of the target object. First, considering the low force of the miniature robot, this 3D structure reduces the effective elastic modulus associated with the effective elasticity, increasing the efficiency of the MR adhesive process due to the low preload force (*50*). The MRE was structured into a 3D mushroom shape using micromolding techniques and stamping processes while uniaxially aligning the fillers (Fig. 4A). The mushroom-shaped structure, considering its columnar geometric design, influences shear adhesion and effective stiffness and its hierarchical structure with tips generates a robust van der Waals force that serves as the source of stable contact with rough surfaces (Fig. 4B). Hence, flat structures limit the effectiveness of their rheological properties because of their strong mechanical resistance forces when applied to rough surfaces, such as bark (root mean square roughness, *R*_q_: 2.9 μm) or pig skin (*R*_q_: 1.27 μm) (SEM images of rough surfaces is shown in fig. S9), showing a wide variation of adhesion with different preloads, as shown in Fig. 4C. In particular, the MR effect is low at low preload, but the effect is more limited on rough surfaces, such as bark. On the other hand, the mushroom-structured adhesive ensures an effective MR effect even on rough surfaces with a low preload (1.5 N/cm^2^), providing robust adhesion performance (magnetic field off: 1.35 N/cm^2^, magnetic field on: 2.93 N/cm^2^) even on very irregular surfaces and effectively controlling adhesion. The analysis of the contact area changes between the adhesive and the rough bark replica during detachment/attachment substantiates the aforementioned process in fig. S10. A clear improvement in adhesion can be confirmed by comparing the area of the contact surface maintained by the magnetic field during preload removal with and without a 3D structure. Second, a critical point of the MR process is that it has limitations in grasping softer materials compared to adhesives. When the substrate, not the MRE, deforms due to the preload, the attractive force of the CIPs only increases the modulus of the MRE but does not restrict the restoration force of the substrate. Ultimately, the persistently high initial modulus of the MRE imposes limitations on grasping softer materials, which can be addressed using the 3D structure. Precision-fabricated 3D structures effectively reduce the effective modulus of the MRE, inducing the deformation of the adhesive rather than the substrate under the same preload conditions. This suggests that 3D structures can efficiently control MR adhesive processes to grasp softer materials. The distinct efficiency of 3D structures for soft and wrinkled targets was verified using adhesion measurements of flat and mushroom-shaped adhesives on rough surfaces with different elastic moduli (Fig. 4, C and D, and stress distribution and contact area analysis with FEM in Fig. 4E). Furthermore, adhesive measurements and FEM analysis confirmed the enhanced adhesion of the mushroom-shaped structure in the shear direction (fig. S11). Therefore, if the amount of moisture is adequate for the capillary force, then MRE adhesion control is possible using an external magnetic field (2 cm by 2 cm, <250 mT field switching). This proves that MRE adhesive control through magnetic fields is suitable even under humid conditions, as shown in fig. S12 and movie S1. Furthermore, the MRE achieves rapid and precise switching of normal and shear direction adhesive, encompassing a dynamic magnetic field (fig. S13).

### Gripping and manipulating objects and tumor removal surgery assistance

**Fig. 5.**
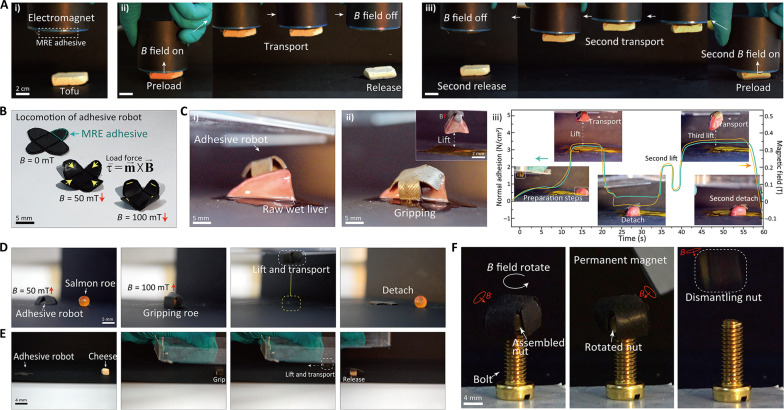
Adhesive soft millirobot demonstrations. (**A**) MRE adhesive gripping, lifting, and transferring soft tofu. (i) Loading MRE adhesive on tofu. (ii) First adhesive control. (iii) Second adhesive control. (**B**) Rolling locomotion of the magnetically programmed soft millirobot, integrated with MRE adhesive under different magnetic fields. (**C**) (i) Raw liver and adhesive robot. (ii) Adhesive robot gripping and lifting a raw liver. (iii) Estimated adhesion force with a theoretical model during the gripping and transferring process. (**D** and **E**) Adhesive robot gripping, lifting, and transferring soft and wet salmon roe and cheese. (**F**) Adhesive robot removing a nut from a bolt by rotating using the trilled magnetic field orthogonal to the *z* axis.

The MRE adhesive was integrated with a soft millirobot that was magnetically controlled to navigate, facilitating versatile manipulation of various objects. Before that, as shown in Fig. 5A, the adhesion control ability of the MRE adhesive was confirmed by manipulating (gripping, lifting, and transporting) moist, soft tofu using only the MRE adhesive without a programmed robot (movie S2). The programmed robot was integrated with this MRE adhesive, and the robot locomotion was designed to be limited at a level similar to the shape of the object and up to a maximum external magnetic field of 100 mT, as shown in Fig. 5B (see Materials and Methods for fabrication details). Figure 5C shows the remote robot manipulating and gripping a wet and soft raw liver sample, one of the ultimate targets that need to be manipulated in biosystems. The robot showed a robust adhesive force even on soft and smooth biological tissue, and the adhesion force in response to the external magnetic field was estimated through a theoretical model during the process [Fig. 5C (iii) and movie S3]. Furthermore, the microrobots are also shown manipulating objects as diverse as smooth, wet salmon roe and soft cheese (Fig. 5, D and E). Beyond simple grasping and carrying, the robot could firmly grasp a nut assembled on a bolt and generate a rotational torque in response to a rotating magnetic field orthogonal to the *z* axis to remove the nut (Fig. 5F).

**Fig. 6.**
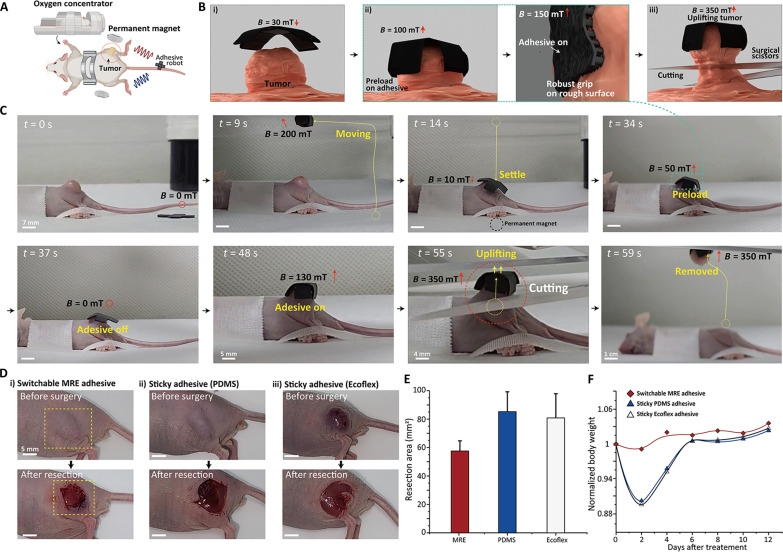
In vivo demonstration of skin tumor removal surgery by the adhesive robot on a mouse. (**A**) Schematic image of a tumor removal surgery of a mouse with an assistant adhesive robot with a magnetic field. This illustration was developed with BioRender.com. (**B**) Schematics of the process of gripping and cutting tumors by a magnetic field. (i) Adhesive robot approaching the mouse tumor. (ii) Gripping the tumor with an enhanced adhesive force by increasing the magnetic field. MRE adhesive conformal contact on the rough and wet tumor with the proper preload force applied using a torque force. (iii) Uplifting the tumor with high adhesive by a magnetic field and cutting the tumor with surgery scissors. (**C**) Sequential depiction of the rapid and precise removal surgery process with controlling the adhesive robot by an external magnetic field. (**D**) Before and after photographs of amputation sites in mice operated using different adhesive-based robots. (**E**) Arithmetic values of surgical areas by different adhesives. (**F**) Normalized body weight change in the mouse body after the operation during 12 days after surgery recovery.

The illustration of the in vivo experiment in Fig. 6 and movie S4 shows the capability of using the adhesion control as a critical tool in the remote removal surgery process and the locomotion of the magnetically programmable soft robot with MRE adhesive patches (Fig. 6A). Tumor removal stands as a pivotal means to eliminate tumors, but difficulties arise in surgery when tethered tools cannot reach the location. In addition, tumor cells, with their exceedingly soft and wrinkled surface properties, make it difficult to grip because of their small diameter of approximately 3 to 7 mm without damage. The adhesive robot overcame these limitations, enabling smooth and precise adhesion suitable for gripping tumors for removal. Figure 6B presents a simplified schematic image of the specific tumor grasping and cutting process of the robot. Figure 6C shows the sequential procedure of remotely removing tumors implanted in a mouse leg using the MRE adhesive robot, suggesting its suitability for in vivo applications. As previously mentioned, while the robot movements can also be induced by external magnetic fields of up to 100 mT, the MRE adhesive is activated in magnetic fields exceeding this threshold. In this process, the robot movement and gripping controlled by adhesive force are free, allowing precise gripping only at the desired target, and as a result, the adhesive robot can precisely grab and lift tumor cells and remove only the part that needs to be cut. Furthermore, by comparing the surgical progress made by robotics equipped with nonswitchable adhesives (pure PDMS and pure Ecoflex) to the area of incision (Fig. 6, D and E) and the changes in the mouse’s weight (Fig. 6F), we reinforce the importance of switchable adhesive with precise remote surgery. Consequently, the mouse’s tumor ablation experiments using magnetic force–based remote adhesion switching show that it is possible to firmly grip and move somatic cells through precise, gentle, noninvasive detachment/attachment without harming the biological system. Previous grippers have been difficult to fabricate owing to material and spatial limitations, rendering them unsuitable for applications in small-scale environments such as the in vivo scenario. All demonstrations and results in Fig. 6 suggest the potential for adhesive robots to serve as cellular grippers for surgery within the body, initiating the possibility of remote surgeries in difficult-to-access internal spaces where tethered devices struggle to enter.

## DISCUSSION

This study developed and tested a miniaturized and magneto-programmable adhesive inspired by an organism that transforms the mechanical properties of its secretion. This soft adhesive, fabricated as a 3D structure, controls the gripping forces with variable stiffness (modulus ranging from 0.477 to 2.03 MPa in single material composition), with a delicate and firm grip on diverse surfaces by a low preload. The remotely controlled soft millirobot, integrated with the developed adhesive, can move freely and quickly control attachment remotely by controlling the adhesive with a magnetic field that does not harm living tissues. Last, the adhesive robot can be applied to remote tumor removal surgery with the capability to improve conformal contact even on biological cell surfaces with severe wrinkles. Thus far, there has been little research on remotely controlled adhesion, and the friction and adhesion on the robot surface have been perceived merely as a means of locomotion, not as a control element.

### Material optimization

The current study focused on the change rate of adhesion and optimizing the material by investigating the change rate of modulus and surface energy caused by the magnetic field according to the composite composition ratio. Although the most commonly used materials have been optimized, there is a need to develop softer materials with stronger mechanical deformation to resolve the mechanical mismatch between the internal organs and the adhesive robot interface. The scalability of adhesion control by this process can be increased by developing softer materials with a higher permeability of MR particles because of the ineffective MR adhesive process on softer objects than the adhesive. Furthermore, the current MRE has a limitation in that the stiffness can only be increased by an external energy source, necessitating continuous external energy (magnetic field). The limitation of the need for a continuous external energy source can be overcome by developing a material in which the storage modulus can be decreased using an external energy source. In addition, our fabrication process did not address material improvement through chemistry, as it is based on a simple mixing process. Several studies have been published on the performance (composition ratio and modulus change) of composites through coating particles and surface treatment in solution processes (*51*, *52*). We anticipate that integrating these studies into our ongoing research will further advance the research field of MREs in the future.

### Enhanced adhesion forces through theoretical mechanism

This section starts with an analysis of the theoretical model of the adhesion control process. First, the vertical adhesion force (*F_N_*) was calculated according to the existing literature (*53*).

$$F_{N}=k\sqrt{\frac{6A\pi \gamma a}{C}}$$ (2)

where *A* is the contact area between the surface and the adhesive, *a* is the length along the contact area, γ is the work of adhesion of the interface, $C(\frac{1}{E})$ is the adhesive compliance, and *k* is the proportionality constant. When investigating adhesion on a flat substrate, the main factors, γ and *C*, which change with the external magnetic field, were obtained by approximating the experimental values analyzed in Fig. 2 (D and F) (see figs. S14 and S15 for approximations). Assuming a proper preload for a flat substrate, changes in other factors except for elastic modulus and work adhesion of the interface are irrelevant, and the adhesion can be predicted simply using Eq. 2. On a rough surface, however, the adhesion process needs to be tracked step by step. An analysis of each element’s value is necessary, including the initial area, the polymer’s restoration force constrained by CIP attractive forces, the resulting changes in contact area, the variation of MRE modulus due to CIP attractive forces, and the total net force (adhesion force) caused by these changes. The initial contact area (*A*_0_) can be obtained by FEM analysis with preload (*F_p_*) and elastic modulus by measuring the roughness by SEM (fig. S9). As shown in fig. S9, the pig skin surface has a Gaussian height probability distribution with a root mean square (*R*_q_) roughness of 1.27 ± 0.1 μm and with the highest point ∼29 μm above the average plane (*54*). In the next step, when the magnetic field is applied during the preload, the adhesive force (*F*_*N*, *M*_) due to the initial contact area and the changed modulus is as follows

$$F_{N,M}=k\sqrt{\frac{6A_{0}\pi \gamma_{m}a}{C_{m}}}$$ (3)

The preload is limited to a maximum up to the value where the initial area is saturated. In addition, under the application of a magnetic field, the inter-CIP attraction suppresses the polymer resilience after the preload is removed. The restoring force (*F_r_*) is similar to the preload force

Fr≈Fp (4)

Here, the maximum preload force is limited to the value at which the contact area is saturated. The attractive forces between CIPs acting on the polymer (*F_a_*), which limits the resilience of the deformed polymer, can be obtained as a function of the composition ratio and the applied magnetic field (Fig. 3, D and E) (*55*). The interaction force (*F*_dip_) of the particles as a point dipole, dispersed inside the cube (*d*^3^, 50 μm by 50 μm by 50 μm), is obtained by changing the composition ratio of the MRE (distance of particles) by FEM analysis and equations (note S2). The attractive force per unit area (*F_a_*) of the MRE can be expressed as

$$F_{a}=\frac{F_{\text{dip}}}{d^{2}}$$ (5)

The adhesion net force *F_N_* in the MRE adhesion process on the rough surface is expressed as follows

$$F_{N}=F_{N,M}-F_{r}+F_{a},\left(F_{r}>F_{a}\right)$$ (6)

$$F_{N}=F_{N,M}..\left(F_{r}<F_{a}\right)$$ (7)

In addition, the MRE adhesive was designed with a mushroom-shaped structure to enable conformal contact over a wide surface area on a wrinkled surface with a low preload force. This study considered the weak preload force to be an important factor because the low torque force of the robot may limit the adhesive structure. The theoretical adhesion force was also estimated on the basis of the changes in the adhesive elements caused by the external magnetic field shown in Fig. 2. The factors that change are the contact area and surface energy due to the structure. By modifying Eq. 2, the adhesion due to contact after preload can be expressed as

$$F_{N,M}=k\cdot n\sqrt{\frac{6A_{0,m}\pi \gamma_{m}r}{C_{m}}}$$ (8)

where *A*_0, *m*_ is the contact area between the surface and one of the mushroom structures, *r* is the radius of the flat tip, and *n* is the number of pillars. For adhesion in relation to softness, the modulus acting on adhesion was calculated using the relative effective modulus (*56*) and applied to the adhesion equation (Eq. 7).

In mechanical adhesion mechanisms, however, a simple surface area increase, preload force relaxation, and leverage-specific MR responsiveness can be applied. For example, altering the composition ratio of the MRE structure markedly reduces the surface energy due to mechanical deformation. Exploring the synergy between such mechanical deformations and structures provides an opportunity for applied research to control and enhance adhesion. In this regard, future studies will develop new structures to enable in vivo applications, the ultimate goal of remote robotics. Based on this, the development of robots powered by mechanically deformable adhesion mechanisms could be a powerful future alternative in the body, a harsh environment with constant fluid secretion.

### Application design

This robot was used to perform remote tumor removal surgery on a mouse leg. Its crucial role included grasping and lifting the tumor to minimize the cutting area. The robot effectively gripped and lifted tumor cells on slippery and rough surfaces, enabling precise removal surgery. Nevertheless, the experiments were conducted on remote tumor removal surgery in vitro rather than in vivo because of the limitations and the required equipment like a tracking system and remote cutter. Currently, the cutting method using scissors is widely used in robot surgical incisions (*57*). Other cutting methods, such as hyperthermia, lasers (*58*), magnetic field remote scissors (*59*), wires (*60*), and ultrasound (*61*), have been introduced, and our adhesive robotic technology could be integrated with these cutting methods in the future.

In addition, there are systemic limitations to a magnetic system. Currently, devices like the Helmholtz coil, which can control the magnetic field precisely, face challenges in forming a uniform magnetic field of over several hundred millitesla, except for MRI equipment. The ideal conditions for surgery and actual operations exist in such environments. Experiments using permanent magnets were conducted owing to the limitations of the weak magnetic field size inherent in the coil. Even when considering the potential downsizing of future small adhesive robots, powerful magnetic field control devices are essential for delicate and precise manipulation within the human body, enabling tasks such as gripping and specific position fixation. A future soft gripper with all these adjustment capabilities could expand applications in the field of biomedical engineering, such as in implantable closed-loop operating systems.

## MATERIALS AND METHODS

### Fabrication of 3D structured MRE adhesive

The base material of MRE adhesive is a polymer composite containing CIPs (diameter: ~3 μm, density: 3.9 to 4.3 g/cm^3^, BASF SE, Germany) with soft magnetic properties and high permeability mixed at a mass ratio of 1:1 to 1:4 (matrix: Ecoflex-10 matrix, Smooth-On Inc.; density: 1.04 g/cm^3^). The adhesive in three dimensions was structured by casting an acrylic mold with a 3D hole structure and producing a pillar structure using the molding process. The acrylic mold can be made in various sizes, but the optimized scale (diameter: 100 μm, height: 200 μm, space ratio: 0.7) was made by referring to existing papers. During the molding process, the MRE composite is cured by applying an appropriate magnetic field (50 mT) in the vertical direction for anisotropic alignment of the fillers inside the matrix (fig. S3). To prevent the sedimentation of the magnetic particles during the curing process, we rapidly cure the polymer through heat treatment after sufficient mixing and sonication. The structured MRE exhibited enhanced magnetic responsiveness owing to its directional and high-permeability filler. Last, a mushroom-shaped structure was achieved using a stamping process to attach tips to pillar structures. Similarly, an appropriate magnetic field (100 mT) was applied while curing the composite within the tip structure.

### Investigating and optimizing MRE adhesive properties

Load/force measurement equipment (Instron, 68SC-2, MA, USA) and a custom-made magnetic field application device were used to measure the change of the elastic modulus of the MRE arranged with anisotropic or isotropic fillers. This custom device (KB Magnet Co. Ltd., Republic of Korea; details in figs. S16 and S17) minimizes MRE deformation by gradient forces and only applies a uniform magnetic field. The modulus change of a material was estimated by measuring the stresses applied to a deformed MRE with different magnitudes of a uniform magnetic field. In this process, the composition ratio of the MRE was optimized to obtain the maximum modulus change rate of the material. In addition, it is necessary to measure the surface energy, which is an important factor for adhesion. The surface energy was calculated by measuring the water contact angle and glycerol contact angle of the MRE samples. In addition to measuring the surface energy of the materials according to the composition ratio, the change in the surface energy due to the external magnetic field was measured, and the composition ratio was optimized to maximize the adhesion force change using the two factors (surface energy and elastic modulus). The adhesion force with the actual force required while attaching and detaching the MRE adhesive to various target samples (silicon wafer, Dragon Skin, and Ecoflex) was measured, including theoretical modeling of the adhesion force predicted by the two factors investigated earlier, and the results were compared.

### Cell culturing

The human colon adenocarcinoma cells HT-29 (American Type Culture Collection, Manassas, VA, USA) were cultivated with RPMI 1640 medium (WELGENE, Gyeongsangbuk-do, Republic of Korea) containing 10% fetal bovine serum (Gibco, NY, USA) and 1% antibiotic-antimycotic (Gibco, NY, USA) at 37°C in a humidified atmosphere containing 5% CO_2_.

### Preparation of the tumor-bearing mouse model

Eight-week-old male BALB/c nu/nu mice (Orient Bio, Gyeonggi-do, Republic of Korea) were bred under pathogen-free conditions at the Korea Institute of Science and Technology (KIST). All experiments with live animals were performed in accordance with the relevant laws and institutional guidelines of the Institutional Animal Care and Use Committee (IACUC) in the KIST, and IACUC approved the experiment (approval number 2022-021). The xenograft mouse models were prepared by inoculating 1 × 10^7^ HT-29 cells in 100 μl of phosphate-buffered saline subcutaneously into the flank of the mice. The adhesive robot test was conducted when HT29 tumor volume reached 200 to 250 mm^3^. The tumor volume was measured using calipers, and tumor volume (in cubic millimeters) was calculated as 7 mm by 7 mm by 5 mm.

### Magnetically programmable soft millirobot

The base material of the soft robot was a composite polymer composed of a suitable ratio (1:1.5) between the soft polymer PDMS matrix (Dow Corning, SYLGARD 184 Silicone Elastomer Kit) and NdFeB particle fillers (Magnequench, MQP-15-7; average diameter: 5 μm; density: 7.61 g/cm^3^), which has a high residual magnetic flux density and a high coercivity. The high residual magnetic flux density can generate motility in a soft robot with effective magnetic responsiveness, and the high magnetic coercivity ensures that the magnetization profile of the robot is maintained during external magnetic operation. The robot was constructed by pouring the uncured composite polymer into a mold manufactured using a 3D printer and cast. The robot mold was fabricated in a shape that properly grips the tumor cell. The robot was programmed by wrapping its soft body around a 3D-printed structure modeled after the tumor cell and exposing it to a large, uniform magnetic field of 1.4 T (fig. S18). This soft robot, programmed to match the shape of the cell, was motile up to a magnetic field of approximately 100 mT, and its motility at higher magnetic fields became saturated. Consequently, the MRE adhesive was incorporated into this magnetized programmed robot to fabricate an adhesive robot that does not affect the robot motility at magnetic fields above 100 mT but only enhances the adhesion of the MRE adhesive.

## Supplementary Materials

### The PDF file includes:

Notes S1 to S4 Figs. S1 to S18 Legends for movies S1 to S4 References
