# Supplementary material for: Stiffness-tunable velvet worm–inspired soft adhesive robot
Source: Sci Adv. 2024 Nov 20;10(47):eadp8260. doi: 10.1126/sciadv.adp8260 (PMC11578180; doi:10.1126/sciadv.adp8260)
Supplement: Supplementary file 1 — Notes S1 to S4 Figs. S1 to S18 Legends for movies S1 to S4 References [file sciadv.adp8260_sm.pdf]

Science Advances
AAAS

Supplementary Materials for

Stiffness-tunable velvet worm–inspired soft adhesive robot

Hyeongho Min *et al.*

Corresponding author: Sungwoo Chun, swchun127129@korea.ac.kr; Metin Sitti, sitti@is.mpg.de

Sci. Adv. **10**, eadp8260 (2024)
DOI: 10.1126/sciadv.adp8260

The PDF file includes:

Notes S1 to S4
Figs. S1 to S18
Legends for movies S1 to S4
References

Other Supplementary Material for this manuscript includes the following:

Movies S1 to S4

Supplementary Text

Note S1. Calculation method for work of adhesion of interfaces.
We can obtain the surface energy using the Owen-Wendt Equation. Use existing surface energy values for liquid-solid interactions (*62*). The followed equations are the first-order system of equations, and to solve it, the surface energy can be obtained by using two liquids of different types. In this study, water and glycerol are used, and the surface energy of MRE can be obtained using the reference value.

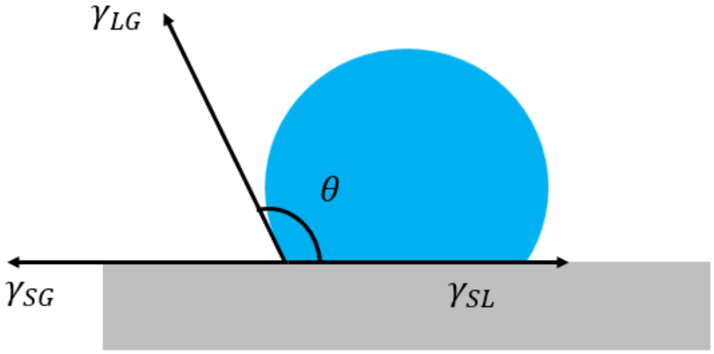

$$Young's\ Equation:\ \gamma_{SG} = \gamma_{SL} + \gamma_{LG}\cos\theta$$

$$Owen - Wendt\ Equation$$

$$\Rightarrow\ \gamma_{SL} = \gamma_{SG} + \gamma_{LG} - 2\left(\gamma_{SG}^{d} \cdot \gamma_{LG}^{d}\right)^{1/2} - 2\left(\gamma_{SG}^{p} \cdot \gamma_{LG}^{p}\right)^{1/2}$$

$$Owen - Wendt\ Equation\ \&\ Young's\ Equation$$

$$\Rightarrow\ \gamma_{LG}(1 + \cos\theta) = 2\left(\gamma_{SG}^{d} \cdot \gamma_{LG}^{d}\right)^{1/2} + 2\left(\gamma_{SG}^{p} \cdot \gamma_{LG}^{p}\right)^{1/2}$$

$$Surface\ Energy:\ \gamma_{SG} = \gamma_{SG}^{d} + \gamma_{SG}^{p}$$

Note S2. Fabrication of aligned CIPs in structured MRE.

To align the CIPs within the MRE along one axis, it is essential to pre-align the fillers before the matrix hardens. To ensure sufficient dispersion of CIPs in the viscous liquid-state MRE, mixing is conducted through a disperser. Subsequently, the mixture is poured into a mold with a hole pattern, and vacuum is applied. During this process, permanent magnets are placed on both sides to apply a magnetic field (50 mT). To minimize the magnetic gradient force acting on the CIPs, a sufficient gap is provided between the MRE-casted mold and the permanent magnets, only allowing for the alignment of CIPs by a uniform magnetic field. For eco-flex, which cures naturally, it can be placed in the vacuum chamber; however, rapid curing can also be achieved in an oven. This process ensures uniform alignment of CIPs in the MRE, promoting optimal conditions for subsequent applications.

Note S3. Analysis of the attractive fore between the CIPs due to the magnetic field and composition ratio.

Once magnetic particles (CIPs) are implanted into the elastomer and external magnetic field was applied, interaction force between magnetic particles will be applied. Under the assumption of magnetic particle as point dipole, the interaction force can be described as following equation;

$$F_{dip} = \frac{3\mu_0}{4\pi}\sum_{\substack{j=1 \\ j\neq i}}^{N}\frac{|m|^2}{|r|^4}\left(\bar{\mathrm{r}}(\bar{m}\cdot\bar{m}) + \bar{m}(\bar{\mathrm{r}}\cdot\bar{m}) + \bar{m}(\bar{\mathrm{r}}\cdot\bar{m}) - 5\bar{\mathrm{r}}(\bar{\mathrm{r}}\cdot\bar{m})(\bar{\mathrm{r}}\cdot\bar{m})\right)$$

Where, μ_0 is air permeability, m is magnetic moment, N is particle number, and r is vector of particle and adjacent particle. $|r|$ is obtained with the composition ratio of MRE. Magnetic moment can be simplify represented as $m = M(H)V$. $M(H)$ is magnetization value in hysteresis graph (Fig. 2C) at magnetic field (H) and V is particle volume.

Herein, we assume the magnetic particles are uniformly dispersed inside the cube (50um × 50um × 50um). The magnetic field is applied in x direction and dipole force of the model was calculated (Fig. S7). As shown in this figure, the force vectors were represented.

Note S4. FEM simulation.

For FEM simulations, a commercial FEM software (COMSOL Multiphysics 5.6, COMSOL Inc.) was used to investigate geometric changes occurring in flat and mushroom shaped architectures under diverse preloads on rough surfaces. In this study, we used a customized two-dimensional model built using model builder, which was automatically meshed using tetrahedral elements. The structural mechanics module was used to analyze the stresses and strains in the structure based on the following equation:

$$\nabla\cdot\sigma + F = \rho$$
$$\varepsilon = \frac{1}{2}[\nabla\mathrm{u} + (\nabla\mathrm{u})^T]$$
$$\sigma = C{:}\,\varepsilon$$

where u denotes the displacement of each point; σ denotes the infinitesimal strain tensor; C denotes the elasticity tensor; and F denotes the external force. For simplicity, pseudostatic analysis and a linear elastic material model were used. For this specific model, the elastic modulus of adhesive was set to 477 kPa and the elastic modulus of substrates was set to 1 MPa and 300 kPa, and Poisson’s ratio was set to 0.5.

Supplementary Figures

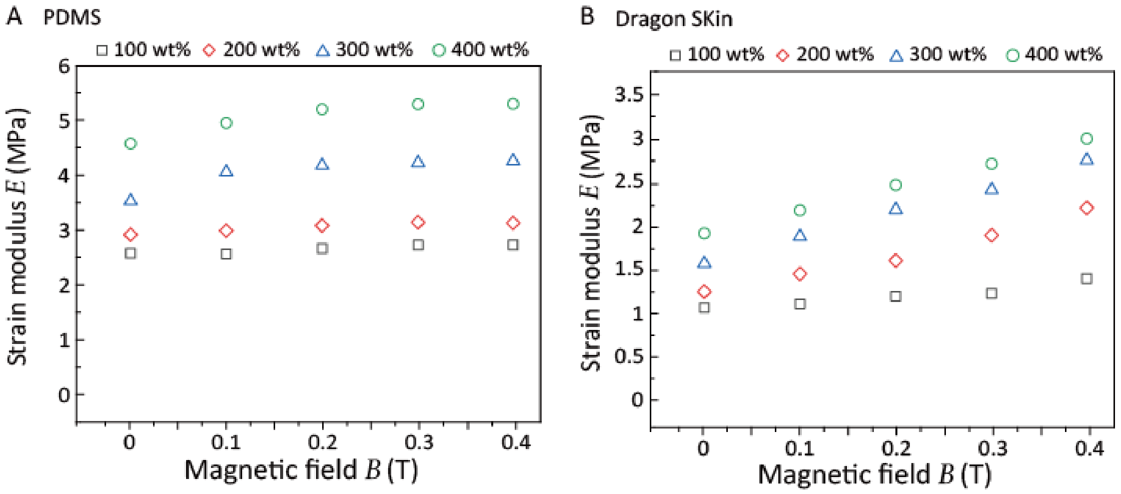

Fig. S1. Mechanical property of magneto-rheological elastomer with high modulus elastomer. (A) Modulus change of CIPs and PDMS composite with different composition ratio, due to external magnetic field. **(B)** Modulus change of CIPs and Dragon skin composite due to external magnetic field.

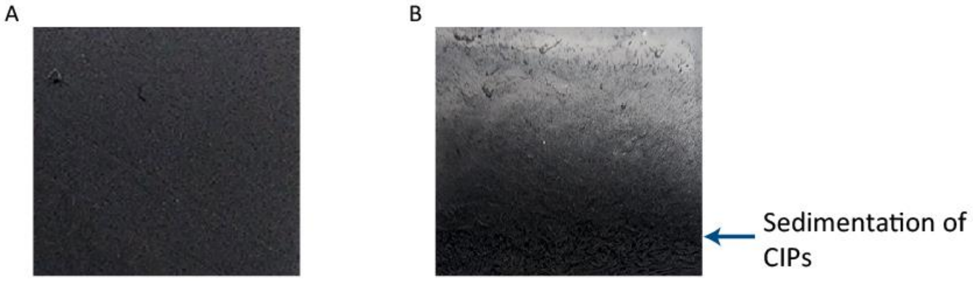

Fig S2. Observation of differences in the sedimentation of CIPs in cross section of the MRE composites which was cured with/out fast heating (A) In 5-minute cured MRE without sedimentation of CIPs. **(B)** Over 20 minutes cured MRE in room temperature with sedimentation of CIPs.

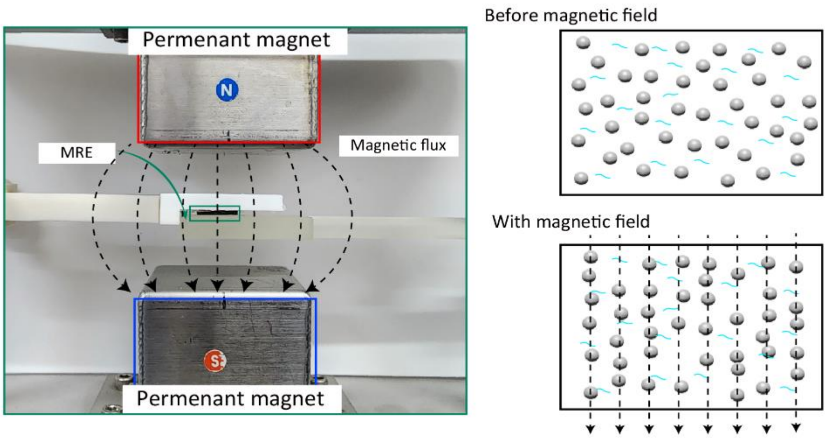

Fig S3. Fabrication process of MRE composite for aligning CIPs. The illustrates demonstrate the alignment of CIPs in MRE with magnetic field

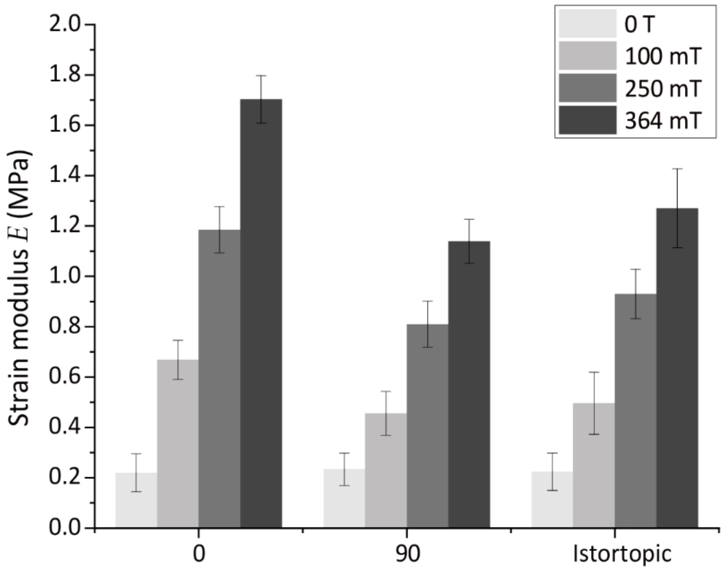

Fig. S4. Anisotropic property of MRE due to its alignment of the fillers. Modulus change of each MRE composite which fillers are aligned in diverse direction, is plotted due to the external magnetic field.

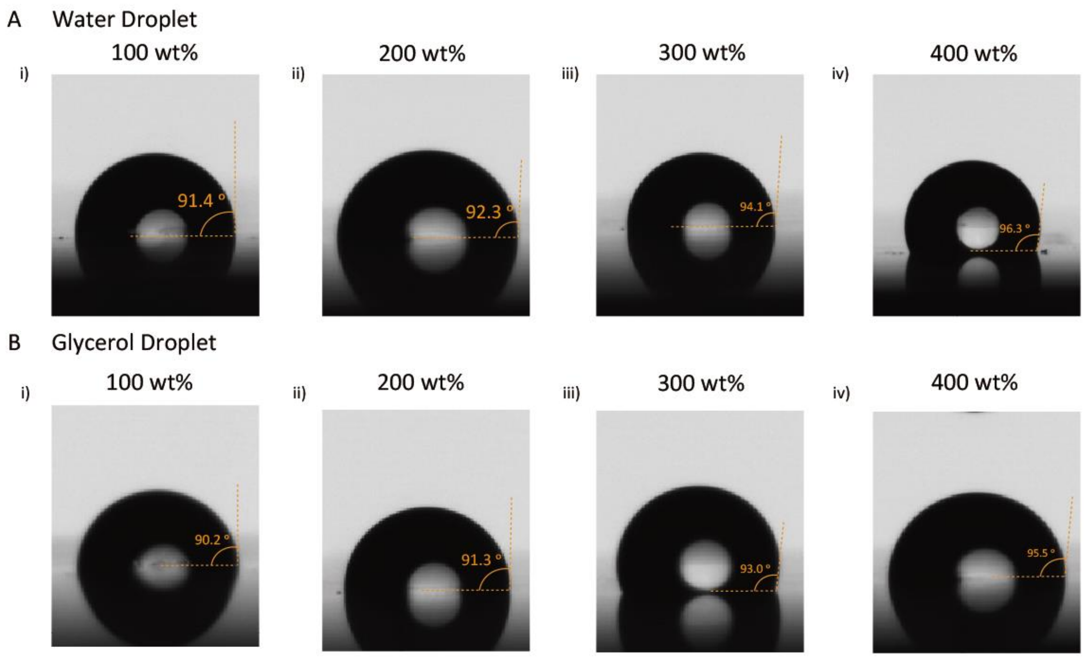

Fig. S5. Contact angle change on the MRE composites due to the composition ratio of the composite. Both contact angle (**(A)**water and **(B)** glycerol) on MRE is investigated for the calculation of surface energy.

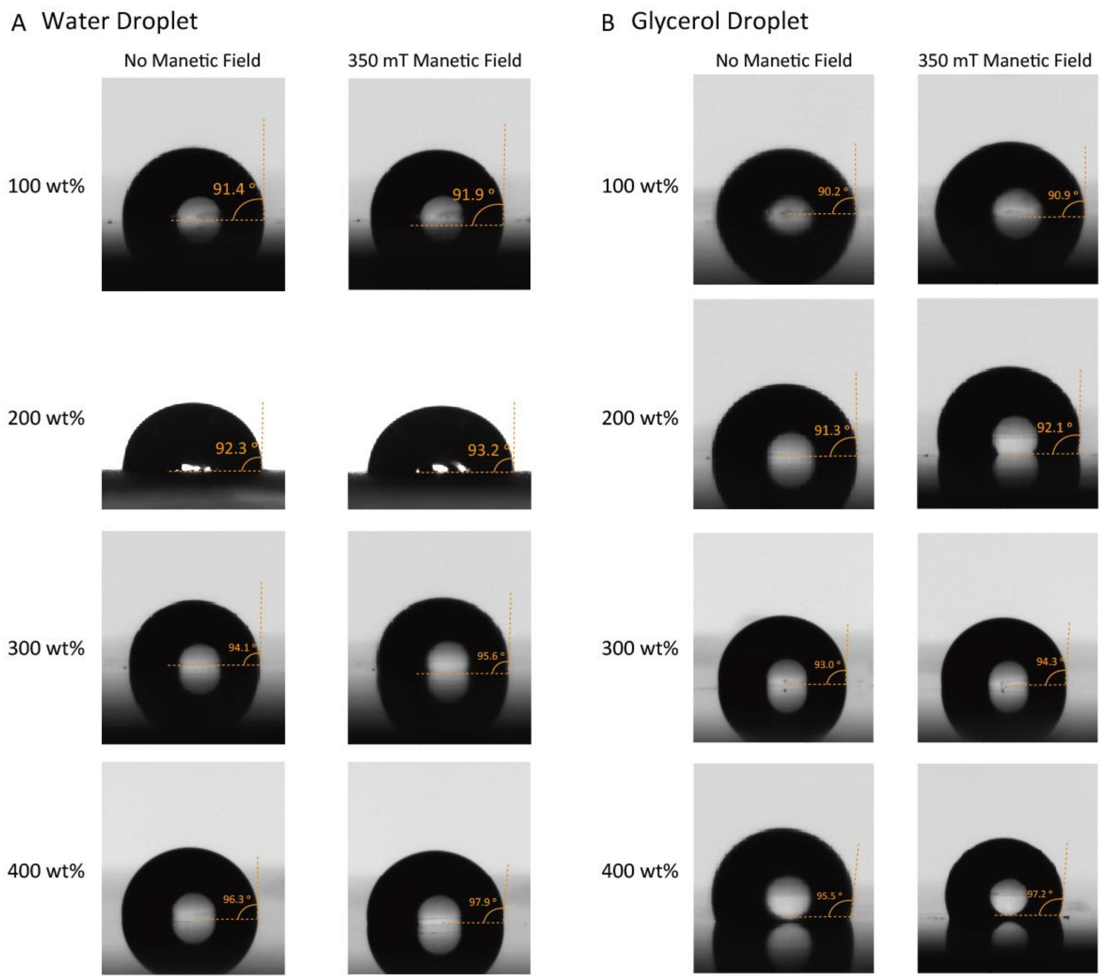

Fig. S6. Contact angle change on the MRE composites due to the change of external magnetic field. Both contact angle (**(A)** water and **(B)** glycerol) on MRE is investigated for the investigation of contact angle change.

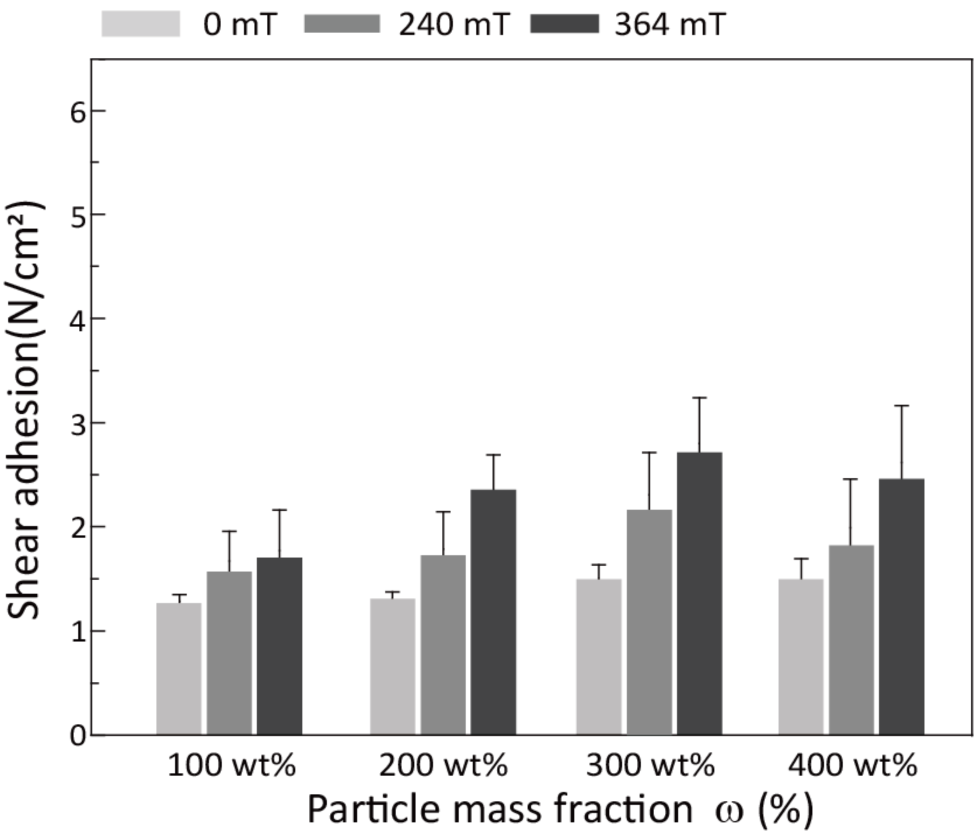

Fig. S7. Analysis of adhesion force of flat MRE adhesive in shear direction due to magnetic field. Shear adhesive is critical elements for the gripping robot, considering the environment where the robots operate.

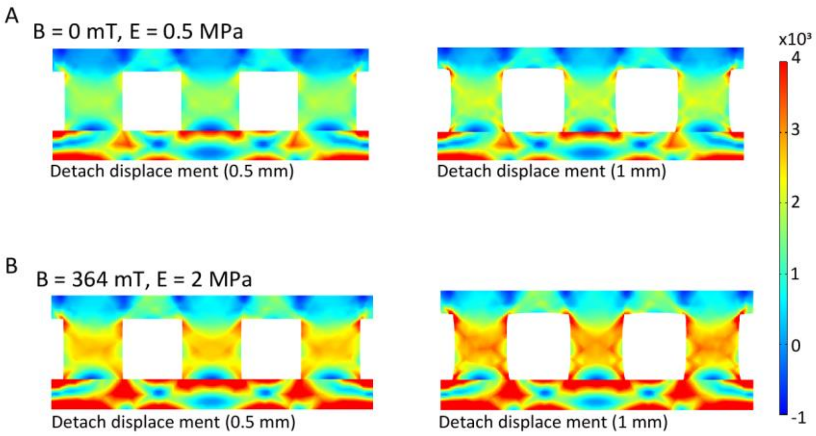

Fig. S8. FEM Analysis of adhesive force due to modulated elastic modulus with the constant contact area. Enhanced mechanical resistance force for pull-off by increased elastic modulus of adhesive material(**(A)** 0.5 MPa and **(B)** 2 MPa).

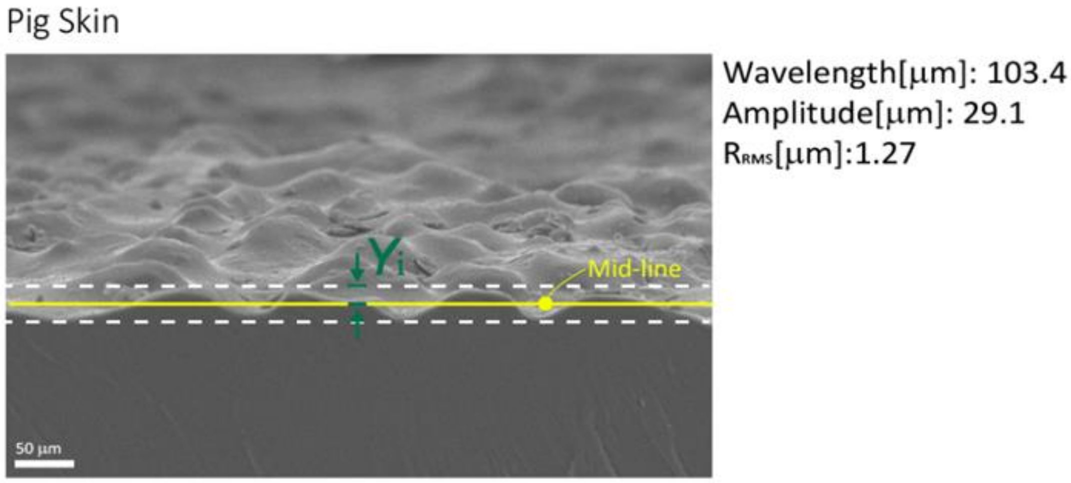

Fig. S9. SEM images of rough surfaces, pig skin. SEM image show the representative cross-sectional image of the surface showing the wrinkle heights ($\boldsymbol{yi}$) and average geometrical values (periodicity, amplitude, and root-mean-square roughness (R_{Rms})) of wrinkles.

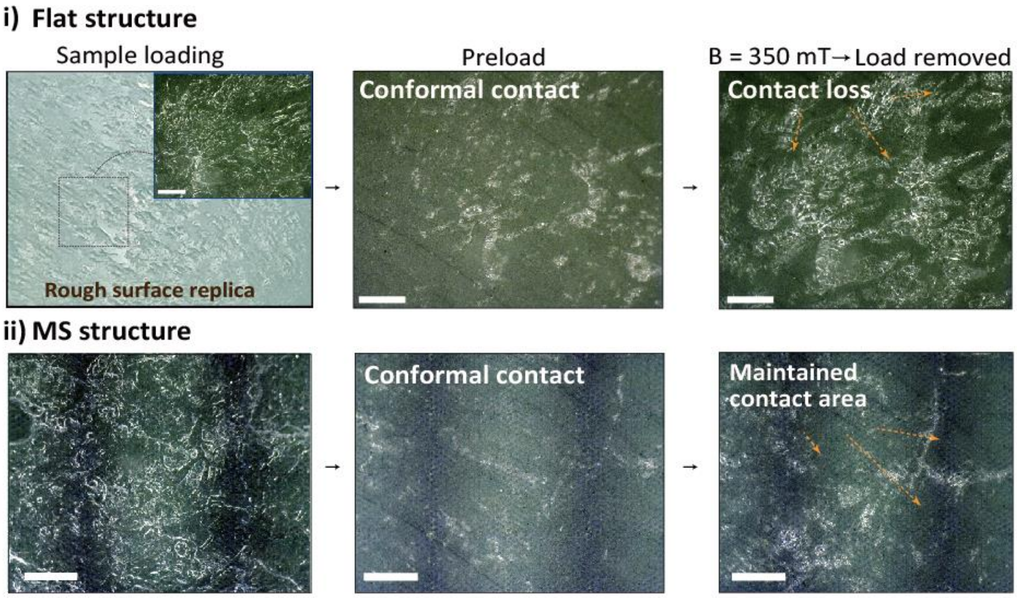

Fig. S10. Differences in contact area change between different structures of MRE adhesives ((i) Flat and (ii) mushroom-shape) to the rough surface. (scale bar: 1 mm)

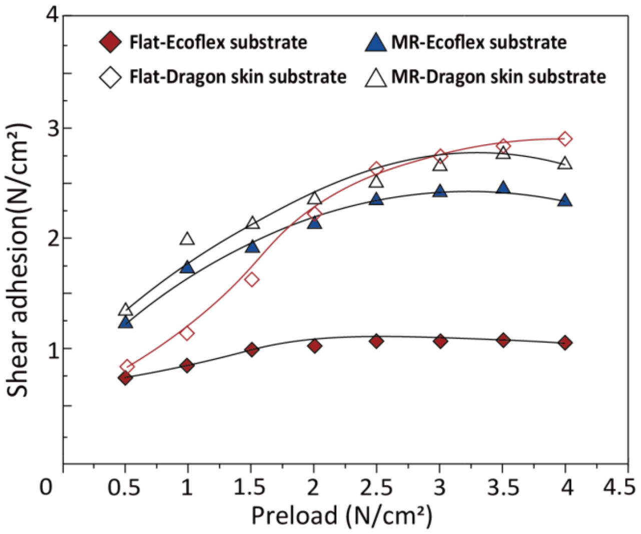

Fig. S11. Adhesive forces measurement in shear direction due to the different elastic modulus of substrate and structure of MRE. Mush room shaped MRE, which is part of pillar structure performs the resistivity against the shear pulling.

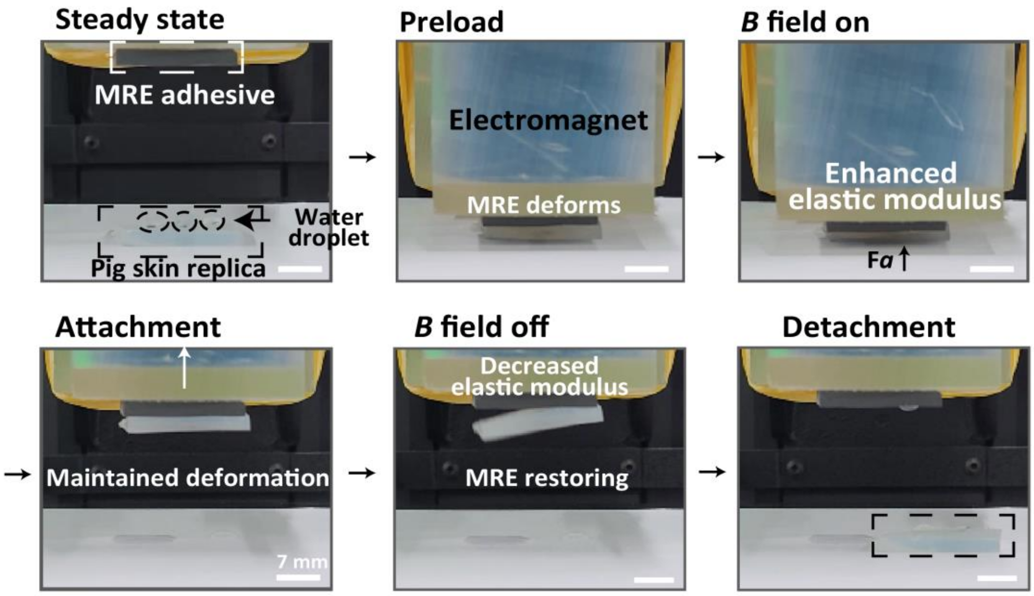

Fig. S12. Sequential image frames showing the capability of adhesion control by an electromagnet in a wet environment.

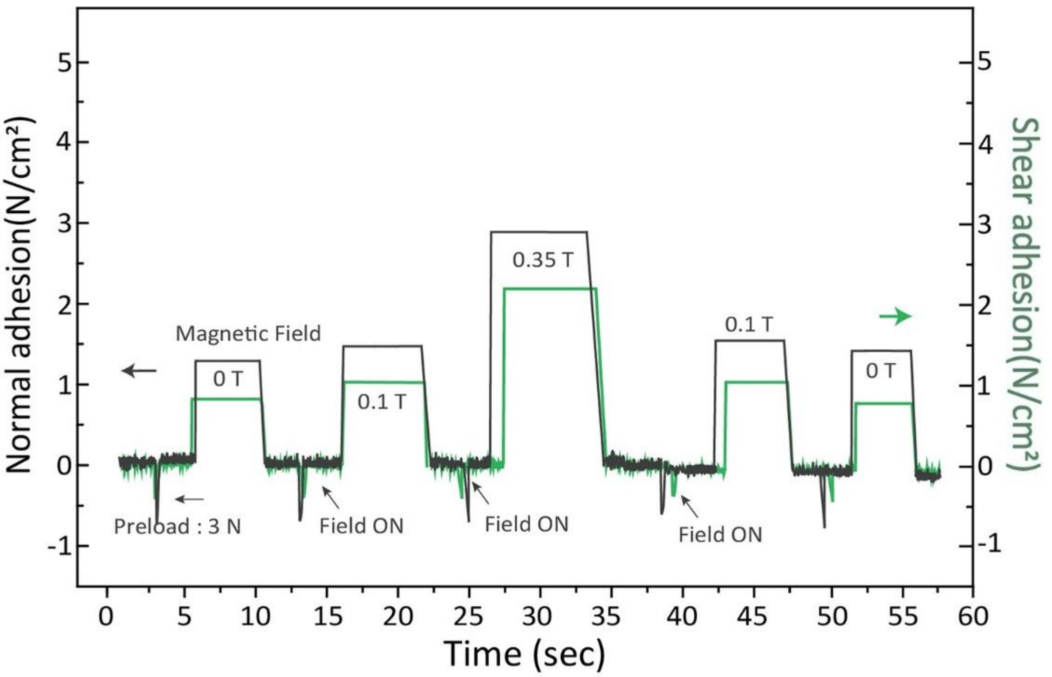

Fig. S13. Measurement of adhesive forces (normal and shear direction) by dynamic change of magnetic field.

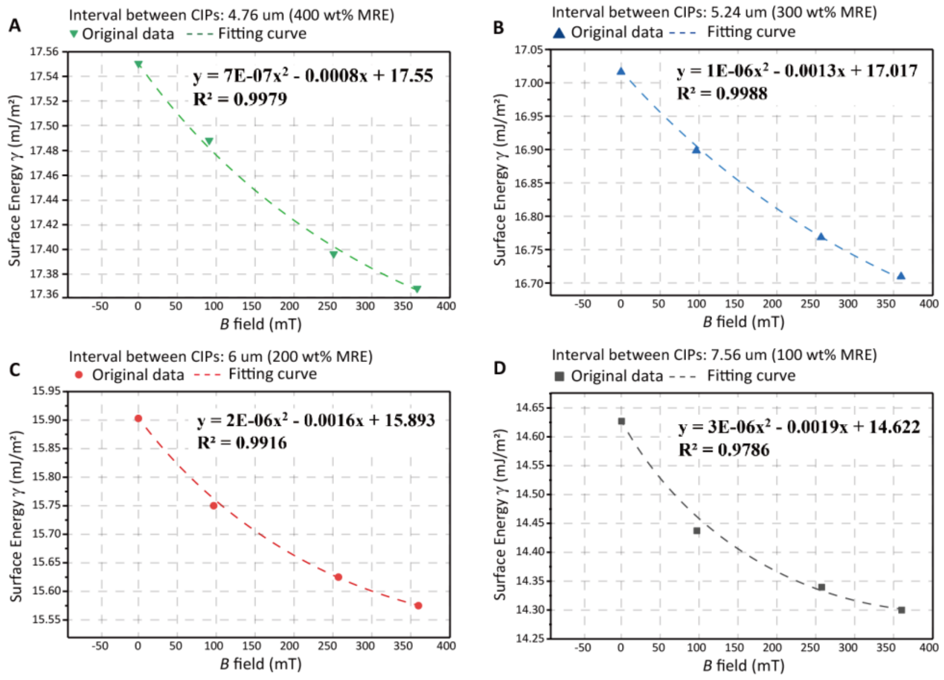

Fig. S14. The approximation of the change in surface energy of different composition ratio of MRE ((A) 400 wt% MRE (B) 300 wt% MRE (C) 200 wt% MRE (D) 100 wt% MRE) due to a magnetic field based on measured data.

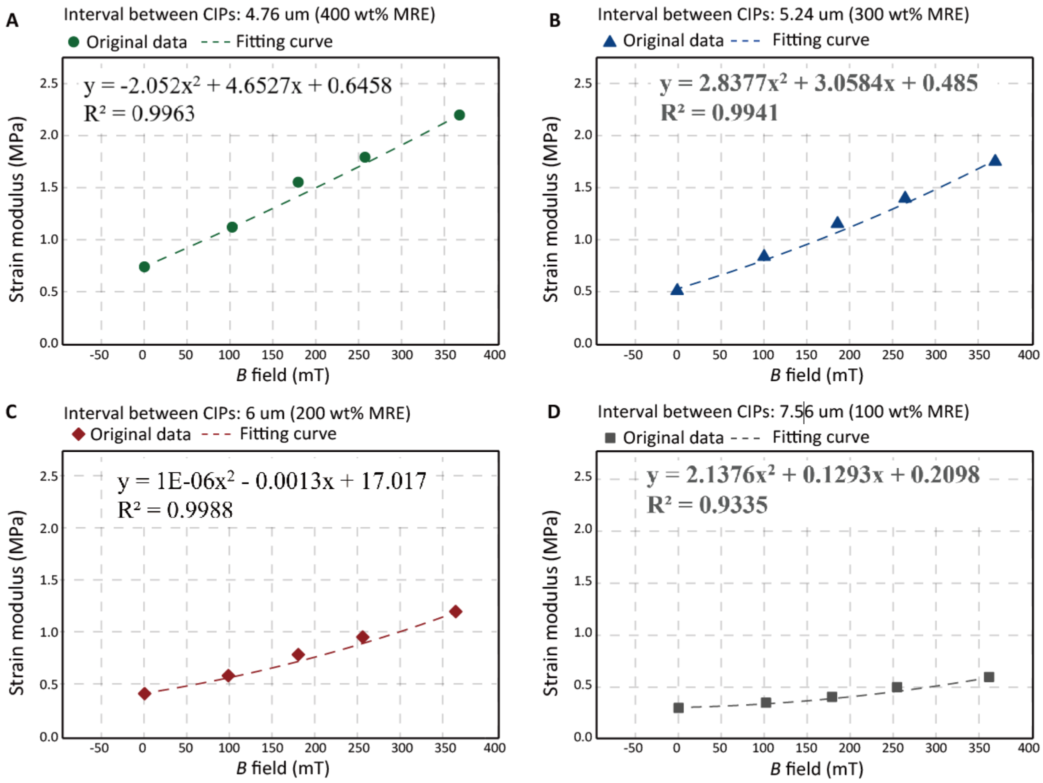

Fig. S15. The approximation of the change in elastic modulus of different composition ratio of MRE ((A) 400 wt% MRE (B) 300 wt% MRE (C) 200 wt% MRE (D) 100 wt% MRE) due to a magnetic field based on measured data.

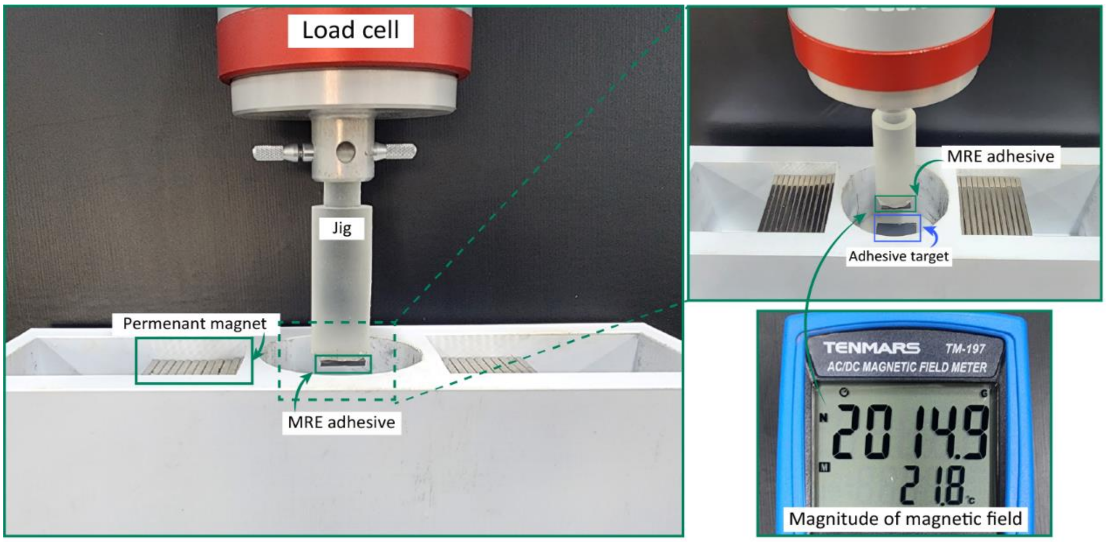

Fig. S16. Measurements of normal-direction adhesion and elastic modulus using load cell and custom-built device. The adhesive sample was attached to the test jig and the target sample was affixed to the bottom of the device.

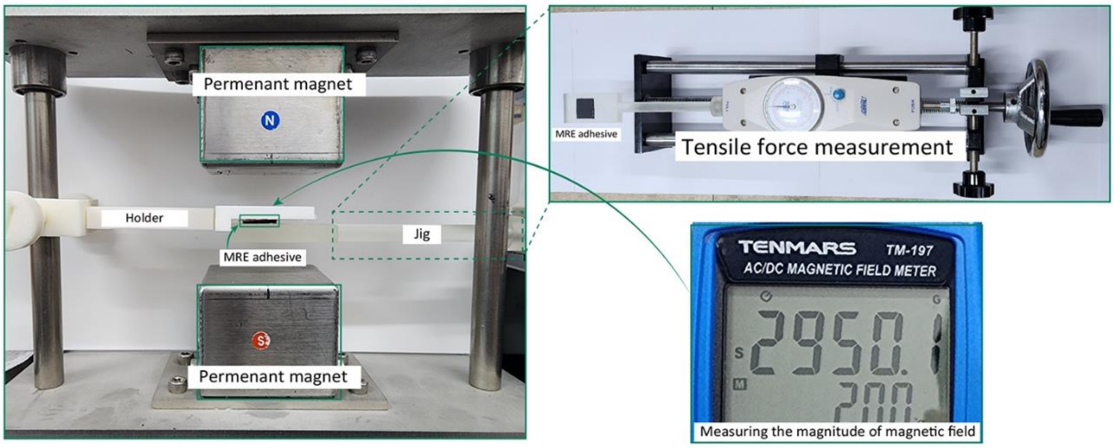

Fig. S17. Measurements of shear-direction adhesion and shear elastic modulus using fore measurement instrument and custom-built device. The adhesive sample was attached to the holder and the target sample was affixed to the jig.

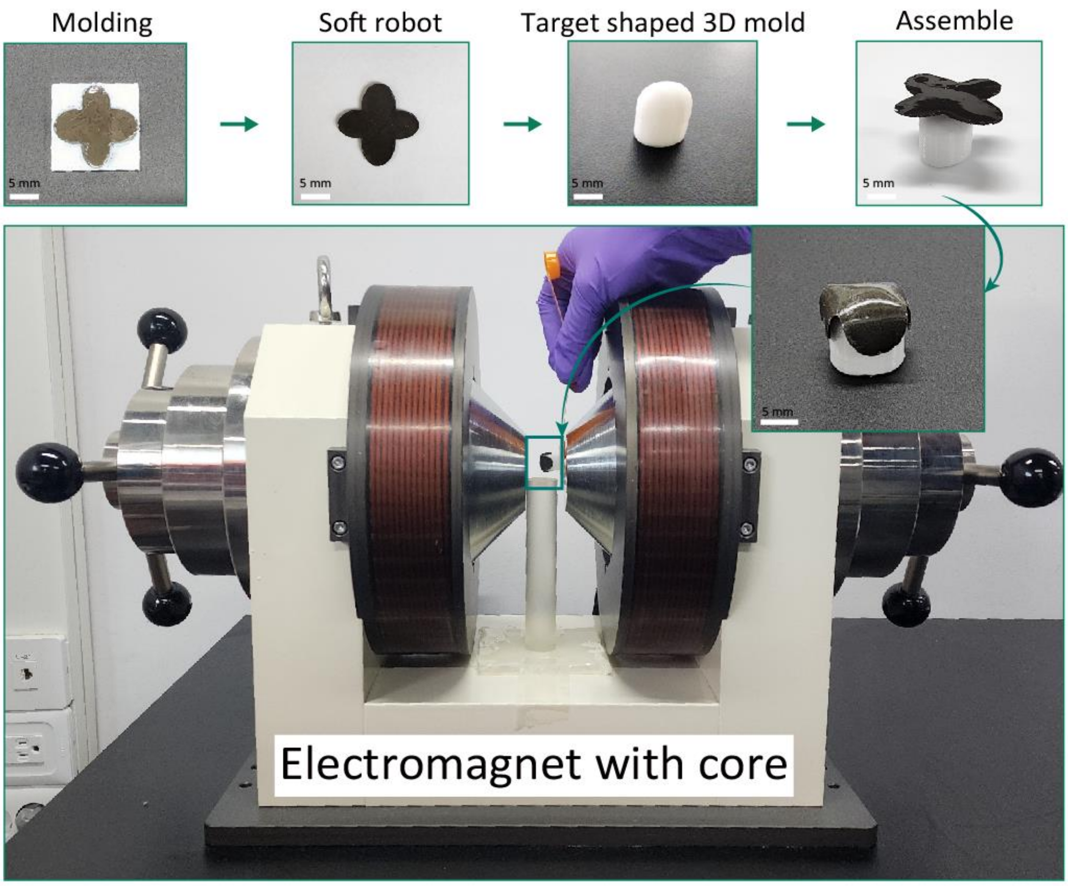

Fig. S18. Electromagnet instrument for magnetization. It is composed with the cores and coils which is surrounding the cores. It applies 1.4 T under each core (distance: 2 cm) by applying 40 A current to coils.

Supplementary Movies

Movie S1. Adhesive control of MRE under humid conditions
Movie S2. Adhesive robot manipulating wet and soft raw liver
Movie S3. MRE adhesive manipulating wet and soft tofu
Movie S4. Mouse tumor removal surgery assistance

REFERENCES AND NOTES

1. M. Sitti, *Mobile Microrobotics* (MIT Press, 2017).

2. B. P. Lee, P. B. Messersmith, J. N. Israelachvili, J. H. Waite, Mussel-inspired adhesives and coatings. *Annu. Rev. Mater. Res.* **41**, 99–132 (2011).

3. C. Menon, M. Sitti, Biologically inspired adhesion based climbing robots. *J. Bionic Eng.* **3**, 115–126 (2006).

4. M. Sitti, R. S. Fearing, Synthetic gecko foot-hair micro/nano-structures as dry adhesives. *J. Adhe. Sci. Technol.* **17**, 1055–1073 (2003).

5. M. P. Murphy, B. Aksak, M. Sitti, Gecko-inspired directional and controllable adhesion. *Small* **5**, 170–175 (2009).

6. S. Kim, M. Sitti, Biologically inspired polymer microfibers with spatulate tips as repeatable fibrillar adhesives. *Appl. Phys. Lett.* **89**, 261911 (2006).

7. G. Huber, H. Mantz, R. Spolenak, K. Mecke, K. Jacobs, S. N. Gorb, E. Arzt, Evidence for capillarity contributions to gecko adhesion from single spatula nanomechanical measurements. *Proc. Natl. Acad. Sci. U.S.A.* **102**, 16293–16296 (2005).

8. K. Autumn, M. Sitti, Y. Liang, A. Peattie, W. Hansen, S. Sponberg, T. Kenny, R. Fearing, J. Israelachvili, R. Full, Evidence for van der Waals attachment for geckos. *Proc. Natl. Acad. Sci. U.S.A.* **99**, 12252–12256 (2002).

9. E. Arzt, S. Gorb, R. Spolenak, From micro to nano contacts in biological attachment devices. *Proc. Natl. Acad. Sci. U.S.A.* **100**, 10603–10606 (2003).

10. D. M. Drotlef, L. Stepien, M. Kappl, W. J. P. Barnes, H. J. Butt, A. del Campo, Insights into the adhesive mechanisms of tree frogs using artificial mimics. *Adv. Funct. Mater.* **23**, 1137–1146 (2013).

11. H. Min, S. Baik, J. Kim, J. Lee, B.-G. Bok, J. H. Song, M.-S. Kim, C. Pang, Tough carbon nanotube-implanted bioinspired three-dimensional electrical adhesive for isotropically stretchable water-repellent bioelectronics. *Adv. Funct. Mater.* **32**, 2107285 (2022).

12. S. Baik, D. W. Kim, Y. Park, T.-J. Lee, S. Ho Bhang, C. Pang, A wet-tolerant adhesive patch inspired by protuberances in suction cups of octopi. *Nature* **546**, 396–400 (2017).

13. S. Li, H. Bai, Z. Liu, X. Zhang, C. Huang, L. W. Wiesner, M. Silberstein, R. F. Shepherd, Digital light processing of liquid crystal elastomers for self-sensing artificial muscles. *Sci. Adv.* **7**, eabg3677 (2021).

14. M. Pilz da Cunha, Y. Foelen, R. J. van Raak, J. N. Murphy, T. A. Engels, M. G. Debije, A. P. H. J. Schenning, An untethered magnetic- and light-responsive rotary gripper: Shedding light on photoresponsive liquid crystal actuators. *Adv. Opt. Mater.* **7**, 1801643 (2019).

15. Y. Roh, M. Kim, S. M. Won, D. Lim, I. Hong, S. Lee, T. Kim, C. Kim, D. Lee, S. Im, G. Lee, D. Kim, D. Shin, D. Gong, B. Kim, S. Kim, S. Kim, H. K. Kim, B.-K. Koo, S. Seo, J.-S. Koh, D. Kang, S. Han, Vital signal sensing and manipulation of a microscale organ with a multifunctional soft gripper. *Sci. Robot.* **6**, eabi6774 (2021).

16. T. N. Do, H. Phan, T.-Q. Nguyen, Y. Visell, Miniature soft electromagnetic actuators for robotic applications. *Adv. Funct. Mater.* **28**, 1800244 (2018).

17. B. Aksoy, H. Shea, Multistable shape programming of variable-stiffness electromagnetic devices. *Sci. Adv.* **8**, eabk0543 (2022).

18. A. G. Gillies, J. Kwak, R. S. Fearing, Controllable particle adhesion with a magnetically actuated synthetic gecko adhesive. *Adv. Funct. Mater.* **23**, 3256–3261 (2013).

19. J. Krahn, E. Bovero, C. Menon, Magnetic field switchable dry adhesives. *ACS Appl. Mater. Interfaces* **7**, 2214–2222 (2015).

20. D.-M. Drotlef, P. Blümler, A. Del Campo, Magnetically actuated patterns for bioinspired reversible adhesion (dry and wet). *Adv. Mater.* **26**, 775–779 (2014).

21. Y. Zhang, W. Zhang, P. Gao, X. Zhong, W. Pu, Finger-palm synergistic soft gripper for dynamic capture via energy harvesting and dissipation. *Nat. Commun.* **13**, 7700 (2022).

22. Z. Zhakypov, F. Heremans, A. Billard, J. Paik, An origami-inspired reconfigurable suction gripper for picking objects with variable shape and size. *IEEE Robot. Autom. Lett.* **3**, 2894–2901 (2018).

23. N. R. Sinatra, C. B. Teeple, D. M. Vogt, K. K. Parker, D. F. Gruber, R. J. Wood, Ultragentle manipulation of delicate structures using a soft robotic gripper. *Sci. Robot.* **4**, eaax5425 (2019).

24. D. Wang, H. Hu, S. Li, H. Tian, W. Fan, X. Li, X. Chen, A. C. Taylor, J. Shao, Sensing-triggered stiffness-tunable smart adhesives. *Sci. Adv.* **9**, eadf4051 (2023).

25. C. Tawk, R. Mutlu, G. Alici, A 3D printed modular soft gripper integrated with metamaterials for conformal grasping. *Front. Robot. AI* **8**, 799230 (2022).

26. H. Tian, X. Li, J. Shao, C. Wang, Y. Wang, Y. Tian, H. Liu, Gecko-effect inspired soft gripper with high and switchable adhesion for rough surfaces. *Adv. Mater. Interfaces* **6**, 1900875 (2019).

27. Y. Hong, Y. Zhao, J. Berman, Y. Chi, Y. Li, H. Huang, J. Yin, Angle-programmed tendril-like trajectories enable a multifunctional gripper with ultradelicacy, ultrastrength, and ultraprecision. *Nat. Commun.* **14**, 4625 (2023).

28. W. Ruotolo, D. Brouwer, M. R. Cutkosky, From grasping to manipulation with gecko-inspired adhesives on a multifinger gripper. *Sci. Robot.* **6**, eabi9773 (2021).

29. S. Song, D. M. Drotlef, D. Son, A. Koivikko, M. Sitti, Adaptive self-sealing suction-based soft robotic gripper. *Adv. Sci.* **8**, e2100641 (2021).

30. C. Linghu, S. Zhang, C. Wang, K. Yu, C. Li, Y. Zeng, H. Zhu, X. Jin, Z. You, J. Song, Universal SMP gripper with massive and selective capabilities for multiscaled, arbitrarily shaped objects. *Sci. Adv.* **6**, eaay5120 (2020).

31. O. Millet, P. Bernardoni, S. Régnier, P. Bidaud, E. Tsitsiris, D. Collard, L. Buchaillot, Electrostatic actuated micro gripper using an amplification mechanism. *Sensor. Actuat. A Phys.* **114**, 371–378 (2004).

32. J. L. Cloudsley-Thompson, *Evolution and Adaptation of Terrestrial Arthropods* (Springer Science & Business Media, 2012).

33. A. C. Noel, H.-Y. Guo, M. Mandica, D. L. Hu, Frogs use a viscoelastic tongue and non-Newtonian saliva to catch prey. *J. R. Soc. Interface* **14**, 20160764 (2017).

34. E. Barajas-Ledesma, C. Holland, Probing the compositional and rheological properties of gastropod locomotive mucus. *Front. Soft Matter* **3**, 1201511 (2023).

35. S. Kim, M. Sitti, T. Xie, X. Xiao, Reversible dry micro-fibrillar adhesives with thermally controllable adhesion. *Soft Matter* **5**, 3689–3693 (2009).

36. A. Baer, S. Schmidt, S. Haensch, M. Eder, G. Mayer, M. J. Harrington, Mechanoresponsive lipid-protein nanoglobules facilitate reversible fibre formation in velvet worm slime. *Nat. Commun.* **8**, 974 (2017).

37. A. Baer, S. Hänsch, G. Mayer, M. J. Harrington, S. Schmidt, Reversible supramolecular assembly of velvet worm adhesive fibers via electrostatic interactions of charged phosphoproteins. *Biomacromolecules* **19**, 4034–4043 (2018).

38. S. Kim, Y. Park, A. Cha, G. Kim, J. Bang, C. Lim, S. Choi, A feasibility work on the applications of MRE to automotive components. *IOP Conf. Ser. Mater. Sci. Eng.* **333**, 012013 (2018).

39. A. Koivikko, D.-M. Drotlef, M. Sitti, V. Sariola, Magnetically switchable soft suction grippers. *Extreme Mech. Lett.* **44**, 101263 (2021).

40. P. Testa, B. Chappuis, S. Kistler, R. W. Style, L. J. Heyderman, E. R. Dufresne, Switchable adhesion of soft composites induced by a magnetic field. *Soft Matter* **16**, 5806–5811 (2020).

41. M. Lanzetta, K. Iagnemma, Gripping by controllable wet adhesion using a magnetorheological fluid. *CIRP Annals* **62**, 21–25 (2013).

42. E. J. Barron III, E. T. Williams, R. Tutika, N. Lazarus, M. D. Bartlett, A unified understanding of magnetorheological elastomers for rapid and extreme stiffness tuning. *RSC Appl. Polym.* **1**, 315–324 (2023).

43. L. Chen, X. Gong, W. Li, Microstructures and viscoelastic properties of anisotropic magnetorheological elastomers. *Smart Mater. Struct.* **16**, 2645–2650 (2007).

44. S. W. Chen, R. Li, Z. Zhang, X. J. Wang, Micromechanical analysis on tensile modulus of structured magneto-rheological elastomer. *Smart Mater. Struct.* **25**, 035001 (2016).

45. K. Johnson, Mechanics of adhesion. *Tribol. Int.* **31**, 413–418 (1998).

46. J. A. Greenwood, K. L. Johnson, The mechanics of adhesion of viscoelastic solids. *Philos. Mag. A* **43**, 697–711 (1981).

47. D. K. Owens, R. Wendt, Estimation of the surface free energy of polymers. *J. Appl. Polym. Sci.* **13**, 1741–1747 (1969).

48. M. D. Bartlett, A. B. Croll, D. R. King, B. M. Paret, D. J. Irschick, A. J. Crosby, Looking beyond fibrillar features to scale gecko-like adhesion. *Adv. Mater.* **24**, 1078–1083 (2012).

49. M. D. Bartlett, A. J. Crosby, Scaling normal adhesion force capacity with a generalized parameter. *Langmuir* **29**, 11022–11027 (2013).

50. M. Schargott, A mechanical model of biomimetic adhesive pads with tilted and hierarchical structures. *Bioinspir. Biomim.* **4**, 026002 (2009).

51. F. Chen, J. Zhang, Z. Li, S. Yan, W. Li, Z. Yan, X. Liu, Effect of the surface coating of carbonyl iron particles on the dispersion stability of magnetorheological fluid. *Sci. Rep.* **14**, 11358 (2024).

52. M. Arslan Hafeez, M. Usman, M. A. Umer, A. Hanif, Recent progress in isotropic magnetorheological elastomers and their properties: A review. *Polymers* **12**, 3023 (2020).

53. Y. Mengüç, S. Y. Yang, S. Kim, J. A. Rogers, M. Sitti, Gecko-inspired controllable adhesive structures applied to micromanipulation. *Adv. Funct. Mater.* **22**, 1246–1254 (2012).

54. L. Dorogin, A. Tiwari, C. Rotella, P. Mangiagalli, B. Persson, Role of preload in adhesion of rough surfaces. *Phys. Rev. Lett.* **118**, 238001 (2017).

55. X. B. Nguyen, T. Komatsuzaki, N. Zhang, A nonlinear magnetorheological elastomer model based on fractional viscoelasticity, magnetic dipole interactions, and adaptive smooth Coulomb friction. *Mech. Syst. Signal Process.* **141**, 106438 (2020).

56. D. Steck, J. Qu, S. B. Kordmahale, D. Tscharnuter, A. Muliana, J. Kameoka, Mechanical responses of Ecoflex silicone rubber: Compressible and incompressible behaviors. *J. Appl. Polym. Sci.* **136**, 47025 (2019).

57. E. Birgin, C. Reißfelder, N. N. Rahbari, Robot with the scissorhands: Scissor hepatectomy for parenchymal transection in robotic liver resection. *J. Gastrointest. Surg.* **28**, 99–101 (2024).

58. J. S. Shin, S. Y. Oh, H. Park, T.-S. Kim, L. Lee, C.-M. Chung, J. Lee, Underwater cutting of 50 and 60 mm thick stainless steel plates using a 6-kW fiber laser for dismantling nuclear facilities. *Opt. Laser Tech.* **115**, 1–8 (2019).

59. O. Onaizah, E. Diller, Tetherless mobile micro-surgical scissors using magnetic actuation, in *2019 International Conference on Robotics and Automation* (IEEE, 2019), pp. 894–899.

60. H. Hammad, B. C. Brauer, M. Smolkin, R. Ryu, J. Obuch, R. J. Shah, Treating biliary-enteric anastomotic strictures with enteroscopy-ERCP requires fewer procedures than percutaneous transhepatic biliary drains. *Dig. Dis. Sci.* **64**, 2638–2644 (2019).

61. O. Nilsson, J. Knutsson, F. J. Landström, A. Magnuson, M. Von Beckerath, Ultrasound-assisted resection of oral tongue cancer. *Acta Otolaryngol.* **142**, 743–748 (2022).

62. A. Rudawska, E. Jacniacka, Analysis for determining surface free energy uncertainty by the Owen–Wendt method. *Int. J. Adhes. Adhes.* **29**, 451–457 (2009).
